# Traffic light labelling could prevent mortality from noncommunicable diseases in Canada: A scenario modelling study

**DOI:** 10.1371/journal.pone.0226975

**Published:** 2019-12-27

**Authors:** Marie-Eve Labonté, Teri E. Emrich, Peter Scarborough, Mike Rayner, Mary R. L’Abbé

**Affiliations:** 1 Department of Nutritional Sciences, Faculty of Medicine, University of Toronto, Toronto, ON, Canada; 2 Nuffield Department of Population Health, University of Oxford, Oxford, Oxfordshire, United Kingdom; University of Rhode Island, UNITED STATES

## Abstract

**Background:**

Traffic-light labelling (TLL) is a promising front-of-pack system to help consumers make informed dietary choices. It has been shown that adopting TLL in Canada, through an optimistic scenario of avoiding, if possible, foods with red traffic lights, could effectively reduce Canadians’ intakes of energy, total fat, saturated fat, and sodium by 5%, 13%, 14% and 6%, respectively. However, the potential health impact of adopting TLL has not been determined in the North American context.

**Objective:**

This study modelled the potential impact of adopting TLL on mortality from noncommunicable diseases (NCDs) in Canada, due to the previously predicted improved nutrient intakes.

**Methods:**

Investigators used data from adults (*n* = 19,915) in the 2004 nationally representative Canadian Community Health Survey (CCHS)-Cycle 2.2. Nutrient amounts in foods consumed by CCHS respondents were profiled using the 2013 United Kingdom’s TLL criteria. Whenever possible, foods assigned at least one red light (non-compliant foods) were replaced with similar, but compliant, foods identified from a Canadian brand-specific food database. Respondents’ nutrient intakes were calculated under the original CCHS scenario and the counterfactual TLL scenario, and entered in the Preventable Risk Integrated ModEl (PRIME) to estimate the health impact of adopting TLL. The primary outcome was the number of deaths attributable to diet-related NCDs that could be averted or delayed based on the TLL scenario compared with the baseline scenario.

**Results:**

PRIME estimated that 11,715 deaths (95% CI 10,500–12,865) per year due to diet-related NCDs, among which 72% are specifically related to cardiovascular diseases, could be prevented if Canadians avoided foods labelled with red traffic lights. The reduction in energy intakes would by itself save 10,490 deaths (9,312–11,592; 90%).

**Conclusions:**

This study, although depicting an idealistic scenario, suggests that TLL (if used to avoid red lights when possible) could be an effective population-wide intervention to improve NCD outcomes in Canada.

## Introduction

Front-of-pack (FOP) labelling is proposed as a public health intervention to improve the dietary intakes and habits of a population, and ultimately its health status [1]. Specific goals of FOP labelling include providing simplified nutrition information to make the healthier choice the easier choice for consumers, and encouraging manufacturers’ reformulation of their food products towards offerings of higher nutritional quality [1]. Research conducted in Europe, Canada and New Zealand suggests that consumers tend to prefer interpretive nutrient-specific FOP systems such as Traffic Light Labelling (TLL) over binary summary indicator systems (e.g. presence or absence of a healthier choice symbol) or information-based systems that only repeat information from the back-of-pack (e.g. percent daily value) [2–5]. Literature reviews and recent studies also suggest that TLL, together with warning labels and the Nutri-Score, are among the most successful types of FOP systems in terms of supporting the identification of healthier food choices [6–11]. In addition, TLL can be easily understood [10], regardless of the nutrition literacy degree of an individual [12], and therefore has the potential to reach the majority of the population.

Public health research is particularly useful for estimating the relevance and effectiveness of nutrition-related policies in order to support their prioritization and adoption by government bodies. Given that randomized controlled trials are not feasible for most policy-based interventions for practical or ethical reasons, scenario modelling is a key tool to assess whether nutrition-related policies can lead to improvements in nutrient intakes and, ultimately, in the health outcomes of a population [13–15]. Current evidence suggests that when TLL is introduced, consumers use it most often to avoid products labelled with red traffic lights rather than increasing their selection of products with primarily green lights [16–20]. Moving away from reds is therefore considered likely to have a greater impact on the consumer than moving, for example, from amber to green lights [16]. In that context, a scenario modelling study has previously shown that adopting TLL, through a strategy of red avoidance, could potentially lead to reductions in body weight and in the number of disability-adjusted life years in the Australian population [14]. Interestingly, another study recently conducted by our group modelled the impact, on the Canadian population’s dietary intakes, of a scenario in which foods labelled with at least one red light were replaced, whenever possible, with similar products from the Canadian food supply without any red light. This optimistic scenario suggested that adopting TLL could be effective in reducing Canadians’ intakes of energy, total fat, saturated fat (SFA), and sodium, as compared to the population’s usual intakes measured in a national nutrition survey [21]. A very recent modelling study similarly showed, although the strategy adopted by consumers in response to TLL was not specified, that TLL could lead to reductions in the intakes of energy, total fat, SFA and salt in the French population [15]. This study also showed that these predicted changes in nutrient intakes could further be translated into a 1.6% reduction in mortality from diet-related chronic diseases [15]. Other than the three above mentioned studies [14, 15, 21], the literature evaluating the impact of TLL on dietary intakes and health outcomes at the population level is scarce, particularly in North America.

Building on our previous work [21], the aim of the present study was to model the potential impact of avoiding foods with red traffic lights on the label on the number of preventable deaths related to non-communicable diseases (NCDs) in Canadian adults, due to the predicted improved nutrient intakes.

## Materials and methods

### Study population

**Fig 1:**
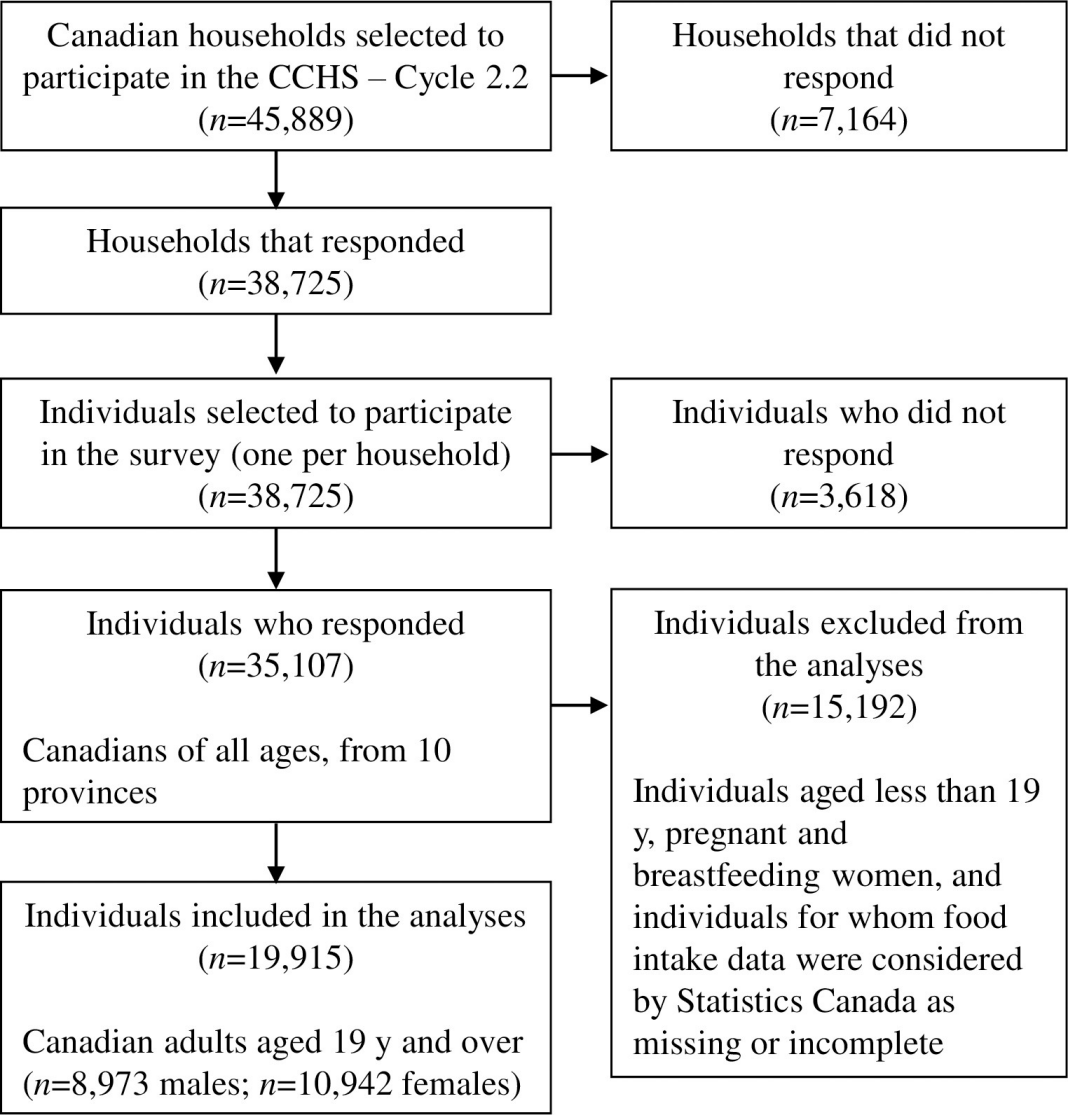
Flow chart of participants in the nationally representative 2004 Canadian Community Health Survey, Cycle 2.2.

The study population consisted of Canadian adults aged 19 years and older from the Canadian Community Health Survey (CCHS)-Cycle 2.2 surveyed in 2004. CCHS 2.2 represented the first national, cross-sectional nutrition survey designed to provide reliable information on the food and nutrient intakes of Canadians since the Nutrition Canada Survey of 1970–72 [22], and the most recent survey available at the time the present study was conducted. Complete details on CCHS 2.2 are available elsewhere [22, 23]. Briefly, the CCHS 2.2 included a total of 35,107 Canadians of all ages from 10 provinces (Fig 1), who lived in private households. Members of the Canadian Armed Forces and residents of Canada’s three northern territories, First Nation reserves or crown lands, institutions, and some remote areas were not included in this sample. The present study also excluded individuals aged less than 19 years, pregnant and breastfeeding women, and individuals for whom food intake data were considered by Statistics Canada as missing or incomplete. Thus, a total of 19,915 Canadians were included in the analyses (*n* = 8,973 men; *n* = 10,942 women).

The current study consisted of secondary analyses of Statistics Canada data, therefore written informed consent was not obtained from participants and it was deemed exempt from an approval by the University of Toronto Health Sciences Research Ethics Board. Statistics Canada and Statistics Canada Research Data Centre guidelines on data confidentiality were followed. All CCHS 2.2 data were anonymized and the research team did not have access to the personal identifiers of participants.

### Dietary data collection

The United States Department of Agriculture Automated Multiple-Pass Method [24], a 24-h dietary recall, was used in CCHS 2.2 to collect information on food consumption. A second 24-h recall was also administered to a subset of the survey participants for estimation of usual nutrient intakes. Nutritional composition information for foods reported in CCHS 2.2 was obtained from the Canadian Nutrient File, version 2001b [25]. The Canadian Nutrient File is Canada’s standard reference food composition database. It is largely derived from the United States Department of Agriculture Nutrient Database for Standard Reference [26], with modifications that reflect specific Canadian fortification and regulatory standards. It also includes some foods consumed in Canada that are not found in the United States database.

### Baseline and TLL scenarios

As previously described by Emrich et al. [21], in 2013, all foods reported as consumed by participants in CCHS 2.2 were first assigned colour codes (red, amber or green) for each of the following nutrients: total fat, SFA, sodium (salt) and total sugars. Colour-coding was based on the criteria detailed in the 2013 version of the United Kingdom’s *Guide to Creating a Front of Pack (FoP) Nutrition Label for Pre-packed Products Sold Through Retail Outlets* [27]. Whenever possible, foods assigned a red traffic light for at least one of the profiled nutrients (non-compliant foods) were replaced with a version of the same product with a more favourable (compliant) nutrient profile, without any red light, in order to create a revised food list. Every effort was made to ensure the replacement food was as similar to the original as possible. For example, the original food could be replaced by the same food of a different brand with a healthier nutrient profile (e.g. President’s Choice brand muesli replaced with Alpen brand muesli; Rogers brand oat flakes replaced with Quaker brand oat flakes). In some cases, the original food was replaced by a version of the same food with a healthier nutrient profile (e.g. lean ground beef replaced with extra lean ground beef, regular cream cheese replaced with fat free cream cheese, salted pretzels replaced with unsalted pretzels). In other instances, foods or recipes were replaced by the same food prepared slightly differently, in a way that yielded a healthier nutrient profile (e.g. beef tenderloin prepared with the fat trimmed to 1/8 inches replaced with beef tenderloin with fat trimmed to 0 inch). A table providing additional examples of substitutions made to create the revised food list are reported in a previous publication [21]. A registered dietitian (TEE) manually identified compliant foods from the Canadian Nutrient File, version 2001b [25] supplemented with data from the University of Toronto Food Label Information Program 2010 [28]. FLIP 2010 is a database with nutritional information on *n* = 10,487 unique branded prepackaged food products with a Nutrition Facts table sold in Canada. If a similar replacement food could not be identified, the non-compliant food was not replaced. Non-compliant foods for which a replacement was not identified comprised, for example, commercial breads for which there was no appropriate lower salt alternative, different types of desserts, candies, and other sweets with no lower total fat, lower SFA and/or lower sugar equivalent (e.g. brownies, certain flavours of ice cream, chocolate covered almonds), different types of processed meats, such as bologna, with no lower SFA and lower sodium alternative, and various nuts and seeds (e.g. almonds, pecans, pistachios) and their butters (e.g. peanut butter) for which there was no lower fat option, although some products were unsalted.

As detailed by Emrich et al. [21] and under Statistical analysis (below), Canadians’ intakes of nutrients of public health concern including energy, total fat, SFA, sodium, and total sugars were then calculated using the unmodified CCHS 2.2 dataset (usual intakes; baseline scenario) and using the revised food list (TLL scenario), without changing the amounts of food consumed. This approach allowed for the examination of the maximum possible impact of the United Kingdom’s TLL system on nutrient intakes, using Canadian-specific composition of foods currently available in the country.

### Health impact modelling

In Spring 2016, the Preventable Risk Integrated ModEl (PRIME) [13] was used to estimate, as a primary endpoint, the total number of deaths attributable to NCDs that could be averted or delayed based on the TLL scenario compared with the baseline scenario. PRIME is a comparative risk assessment model designed to estimate the impact of changes in the age- and sex-specific distribution of one or more out of twelve behavioural risk factors covering diet, physical activity, alcohol consumption and tobacco consumption on NCD mortality, through direct associations or through mediating factors that include BMI, blood pressure and blood cholesterol [13]. Only the sixteen diet-related health outcomes (e.g. hypertension), out of twenty-four possible outcomes, were evaluated in the present study. Links between behavioural risk factors and NCD mortality are parameterized in PRIME based on results of published meta-analyses of epidemiological studies [13]. Risk estimates are combined multiplicatively, and the model was also designed to minimize the risk of double counting of effect size by including parameters which have been appropriately adjusted for other behavioural risk factors. In addition to the total number of deaths related to NCDs prevented by the combined changes in the risk factors of interest, PRIME allows estimations for secondary endpoints including the total number of deaths averted or delayed by sex, and the number of deaths averted or delayed for each cause of death and each behavioural risk factor individually. The PRIME application is available from one of its creators upon request.

The data requirements for the use of PRIME include: 1) age- and sex-specific estimates of the annual number of deaths from each relevant NCD in the population under study; 2) age- and sex-specific estimates of the number of individuals living in the population; 3) the baseline distribution of behavioural risk factors in the population of interest (herein, nutrient intakes under the baseline scenario); and 4) the counterfactual distribution of the variables of interest (herein, nutrient intakes under the TLL scenario). In the present study, data on mortality from diet-related NCDs and population demographics for year 2004 (same year as the dietary intake data) were obtained from the publicly available Statistics Canada CANSIM tables [29–34]. Mortality data, stratified by gender and 5-year age bands, were based on the World Health Organization (WHO) International Classification of Diseases 10 (ICD-10; detailed codes shown in Tables 1 and 2).

**Table 1:** Estimated number of deaths prevented per year under the traffic light labelling scenario, by diet-related risk factor and by cause^a^.

	Deaths averted or delayed					
	Total		Men		Women	
Variables	n (95% CI)	%	n (95% CI)	%	n (95% CI)	%
**Total**^**b**^	11715 (10500, 12865)	100	6699 (6063, 7305)	100	5125 (4339, 5869)	100
**By risk factor**						
Fats (total and SFA)	725 (524, 939)	6	475 (345, 606)	7	254 (178, 345)	5
Salt (sodium)	671 (297, 1079)	6	427 (174, 673)	6	340 (143, 562)	7
Energy balance^c^	10490 (9312, 11592)	90	5934 (5323, 6506)	89	4588 (3828, 5325)	90
**By cause of death (diet-related NCD)**						
Cardiovascular diseases^b^^,^ ^d^	8490 (7442, 9466)	72	4987 (4454, 5514)	74	3622 (2894, 4315)	71
*Ischaemic heart diseases (I20-25)*	5657 (4841, 6405)	48	3814 (3388, 4249)	57	1984 (1360, 2546)	39
*Cerebrovascular diseases (I60-69)*	1336 (866, 1786)	11	581 (388, 766)	9	752 (485, 1010)	15
*Heart failure (I50)*	892 (482, 1222)	8	358 (202, 488)	5	519 (288, 706)	10
*Aortic aneurysm (I71)*	21 (9, 37)	0	16 (7, 27)	0	9 (4, 15)	0
*Pulmonary embolism (I26)*	4 (1, 8)	0	2 (1, 4)	0	2 (1, 5)	0
*Rheumatic heart disease (I05-09)*	2 (1, 5)	0	1 (0, 2)	0	2 (0, 3)	0
*Hypertensive disease (I10-15)*	583 (426, 707)	5	221 (160, 268)	3	356 (267, 426)	7
Diabetes (E11, 14)^d^	1796 (1149, 2241)	15	927 (583, 1164)	14	849 (552, 1050)	17
Cancers^b^^,^ ^d^	960 (733, 1184)	8	484 (374, 595)	7	467 (348, 589)	9
*Colorectum (C18-20)*	512 (350, 675)	4	276 (188, 363)	4	232 (158, 306)	5
*Gallbladder (C23)*	27 (18, 35)	0	10 (7, 13)	0	16 (11, 21)	0
*Pancreas (C25)*	166 (28, 291)	1	82 (15, 144)	1	82 (17, 145)	2
*Breast (C50)*	4 (-72, 77)	0	0 (0, 0)	0	6 (-60, 71)	0
*Endometrium (C54*.*1)*	75 (55, 93)	1	0 (0, 0)	0	68 (49, 85)	1
*Kidney (C64)*	180 (145, 217)	2	117 (93, 141)	2	66 (53, 79)	1
Chronic renal failure (N18)^d^	219 (71, 331)	2	113 (41, 175)	2	103 (35, 154)	2
Liver disease (K70, 74)^d^	293 (86, 463)	3	201 (65, 315)	3	103 (36, 161)	2

^a^Estimates were calculated using the PRIME model [13], based on 2004 Canadian Mortality data [29–34] and on dietary data from the Canadian Community Health Survey, Cycle 2.2 (2004) [22, 23]. NCD, noncommunicable disease; PRIME, Preventable Risk Integrated ModEl; SFA, saturated fat; TLL, traffic light labelling.

^b “^Total” refers to the impact of the combined changes in Canadians’ energy, total fat, SFA and sodium intakes that would result from avoiding foods with red traffic lights on their label. Numbers in the “Total” row represent less than the sums of the individual risk factors and less than the sums of the individual mortality causes since the avoidance of double counting has been accounted for in the modelling process. Similarly, numbers in the “Cardiovascular diseases” and “Cancers” rows represent less than the sums of their respective individual mortality causes since the avoidance of double counting has been accounted for in the modelling process.

^c^ In the PRIME model, energy balance takes into account both changes in energy intakes and changes in physical activity levels. Since physical activity levels remained unchanged between the baseline and TLL scenarios, energy balance here relates solely to energy intakes.

^d^ Codes within brackets are those of the WHO, ICD-10.

**Table 2:** Estimated number of deaths prevented per year under the traffic light labelling scenario, by diet-related risk factor and by cause, excluding the potential impact of changes in the population’s energy balance^a^.

	Deaths averted or delayed					
	Total		Men		Women	
Variables	n (95% CI)	%	n (95% CI)	%	n (95% CI)	%
**Total**^**b**^	1390 (956, 1858)	100	898 (624, 1166)	100	593 (379, 833)	100
**By risk factor**						
Fats (total and SFA)	727 (525, 950)	52	476 (346, 611)	53	254 (180, 344)	43
Salt (sodium)	675 (269, 1087)	49	428 (182, 678)	48	339 (137, 563)	57
**By cause of death**						
Cardiovascular diseases^b^^,^ ^c^	1390 (956, 1858)	100	898 (624, 1166)	100	593 (379, 833)	100
*Ischaemic heart diseases (I20-25)*	1143 (843, 1458)	82	757 (559, 951)	84	445 (324, 575)	75
*Cerebrovascular diseases (I60-69)*	102 (-2, 208)	7	65 (14, 118)	7	55 (-8, 121)	9
*Heart failure (I50)*	55 (22, 92)	4	27 (11, 44)	3	34 (14, 58)	6
*Aortic aneurysm (I71)*	22 (8, 37)	2	16 (7, 27)	2	9 (4, 15)	2
*Pulmonary embolism (I26)*	4 (1, 8)	0	2 (1, 4)	0	2 (1, 5)	0
*Rheumatic heart disease (I05-09)*	2 (1, 5)	0	1 (0, 2)	0	2 (0, 3)	0
*Hypertensive disease (I10-15)*	65 (26, 107)	5	30 (13, 48)	3	45 (17, 78)	8

^a^ Estimates were calculated using the PRIME model [13], based on 2004 Canadian Mortality data [29–34] and on dietary data from the Canadian Community Health Survey, Cycle 2.2 (2004) [22, 23]. The population’s energy balance here solely refers to energy intakes, since physical activity levels remained unchanged between the baseline and TLL scenarios. Diet-related diseases for which there were no changes in the estimated number of deaths averted or delayed (i.e. diabetes [E11, 14], cancers [C18-20, 23, 25, 50, 54.1, 64], chronic renal failure [N18], and liver disease [K70, 74]), were omitted from the table for simplicity. NCD, noncommunicable disease; PRIME, Preventable Risk Integrated ModEl; SFA, saturated fat; TLL, traffic light labelling.

^b “^Total” refers to the impact of the combined changes in Canadians’ total fat, SFA and sodium intakes (i.e. excluding energy) that would result from avoiding foods with red traffic lights on their label. Numbers in the “Total” row represent less than the sums of the individual risk factors and less than the sums of the individual mortality causes since the avoidance of double counting has been accounted for in the modelling process. Similarly, numbers in the “Cardiovascular diseases” row represent less than the sums of the individual mortality causes since the avoidance of double counting has been accounted for in the modelling process.

^c^ Codes within brackets are those of the WHO, ICD-10.

### Statistical analyses

As described elsewhere [21], intakes of energy, total fat, SFA and sodium under the usual (baseline) and counterfactual (TLL) scenarios were calculated for the overall Canadian adult population and for men and women separately, using SAS [35] and SIDE [36] to adjust for day-to-day variation in an individual’s nutrient intake. Bootstrap replication was used for standard error estimations [37] and data were weighted to be representative of the whole Canadian adult population. Mean intakes of energy and nutrients were compared between the baseline and counterfactual (TLL) scenario using a two-sample z-test. Following data entry in PRIME, the model calculated the change in the annual number of deaths attributable to diet-related NCDs between the baseline and counterfactual (TLL) scenarios. The 95% CIs were based on the 2.5^th^ and 97.5^th^ percentiles of results generated from 5000 iterations of a Monte Carlo analysis built in PRIME [13], in which the estimates of relative risks used to parameterize the model were allowed to vary randomly according to the distributions described in the literature. Exploratory sensitivity analyses were also conducted in PRIME, in which the potential impact of changes in energy balance (body weight) on the estimates was removed. Of note, energy balance takes into account both changes in energy intakes and changes in physical activity levels in the PRIME model. Since physical activity levels remained unchanged between the baseline and TLL scenarios, energy balance relates solely to energy intakes in the present study.

## Results

As previously reported by Emrich et al. [21], 52% of 5,655 unique foods and 13% of 495 unique beverages consumed by Canadian adults were assigned at least one red traffic light. Under the optimistic TLL scenario, the percentage of foods and beverages that remained with at least one red traffic light because they could not be replaced by a comparable product without any red light was 40% and 2%, respectively [21]. Canadians’ intakes of energy, total fat, SFA, and sodium under the TLL scenario were 5%, 13%, 14% and 6% lower, respectively (all *P*<0.01), than under the baseline scenario, although intakes of total sugars were unchanged [21].

Of 92,157 annual mortalities in Canadian adults related to the sixteen diet-related NCDs under study (based on 2004 data), 11,715 deaths per year (95% CI: 10,500–12,865; 12.7%) could be averted or delayed through reductions in the intakes of energy, total fat, SFA and sodium resulting from the avoidance of foods labelled with red traffic lights whenever possible (Table 1). More lives would be saved overall among men than women, with 57% of prevented deaths being observed in men. Close to 90% of the reduction in mortality among Canadians would be attributable to changes in obesity status resulting from reduced energy intakes (10,490 prevented deaths; 95% CI: 9,312–11,592) (Table 1).

The majority of potential lives saved would be related to cardiovascular diseases (CVDs) (72%), followed by type 2 diabetes (15%), cancers (8%), liver disease (3%), and chronic renal failure (2%) (Table 1). Similar overall proportions would be observed in men and women separately. Still, in absolute terms, a higher number of deaths related to CVDs would be prevented among men than women. This difference would specifically be driven by changes in ischaemic heart diseases, for which nearly two times more deaths would be averted or delayed in men as compared with women.

Sensitivity analyses showed that only 1,390 deaths per year (95% CI: 956–1,858; 1.5%) could be averted or delayed in Canadians through nutritional changes resulting from the avoidance of foods labelled with red traffic lights that are independent of changes in energy intakes (Table 2). Potential lives saved independently from changes in energy intakes would all be related to CVDs, and more particularly ischaemic heart diseases (82% of deaths averted or delayed).

## Discussion

The present modelling study showed that if food products were to be labelled with TLL and consumers used it to avoid foods labelled with red lights whenever possible, then close to 13% of deaths from diet-related NCDs could be delayed or averted every year in Canada. TLL appears particularly important for the prevention of mortality from CVDs and its potential health impact would primarily be through lower energy intakes among Canadians, corresponding on average to 122 fewer kcal/day consumed by men and 90 fewer kcal/day consumed by women.

In line with the present results, a modelling study conducted in Australia previously estimated that reductions in energy intakes corresponding to approximately 37 kcal/day in men and 21 kcal/day in women resulting from conservative shifts towards the consumption of healthier food choices following the implementation of TLL in selected food categories (i.e., breakfast cereals, pastries, sausages and mixed dishes) would lead to a 1.3 kg reduction in the mean population weight, and subsequently to 45,100 disability-adjusted life years averted [14]. Although the projected reductions in the energy intakes of Australian men and women were 3.3 and 4.3 times lower, respectively, than in the present study, the TLL scenario of Sacks et al. [14] was shown to be both an effective and cost saving public health intervention. A proper cost-effectiveness analysis would be required to confirm whether adopting TLL would also be an effective and cost saving intervention in the Canadian context, but results from Sacks et al. [14] suggest that this strategy appears to be promising. The larger reductions in energy intakes observed in the current study as compared with Sacks et al. [14] also suggest that TLL might be particularly effective if applied broadly across the prepackaged food supply.

One of the reasons why the estimated reduction in mortality from NCDs would primarily be driven by a reduction in energy intakes might be related to the fact that PRIME is very sensitive to changes in energy balance. The model does not account for the possibility of compensatory behaviours following dietary changes (e.g. reduced energy intakes as part of meals might lead to an increase in snacking between meals). This situation explains why sensitivity analyses were conducted to remove the impact of changes in energy balance from the estimations. Our sensitivity analyses can also be seen as an additional counterfactual scenario representing the hypothesized situation in which the expected industry reformulation in response to the introduction of TLL might not induce a change in the energy content of many food products, if the reduction in one or more nutrients labelled in red (e.g. fats) was compensated by an increased content of other macronutrients (e.g. carbohydrates). These analyses showed that without any changes in energy intakes at the population level, the concept of avoiding foods labelled with red traffic lights whenever possible would only slightly reduce mortality from CVDs in Canada (1.5%). Interestingly, this proportion is similar to the one specifically estimated for TLL (1.6%) in the French study which investigated, also using PRIME, the potential impact of different types of FOP designs on reducing mortality from diet-related chronic diseases, although the observations from that French study do include the predicted changes in energy intakes (-6.4%) [15].

Hawkes et al. [38] have recently proposed that the most effective food policy actions are those that are shaped on a population’s preferences and behavioural, socioeconomic, and demographic characteristics. With regards to food labelling, Canadian consumers have already expressed their preference for nutrient-specific approaches such as TLL over summary indicator systems and also showed they could correctly interpret the meaning of the TLL colours [2]. Still, Health Canada recently proposed the mandatory application of a somewhat different nutrient-specific FOP labelling approach, requiring the identification of foods “high in” SFA, sodium, and/or sugars [39]. Avoiding foods carrying “high in” labels could be considered, at least in part, as paralleling the TLL strategy modelled in the present study, which consisted of replacing foods labelled with at least one red traffic light by comparable foods without any red light, if possible [16–20]. Interestingly, the proposed Canadian criteria for “high in” labels appear similar to or stricter than the United Kingdom’s criteria for a red traffic light in many categories of prepackaged foods (e.g. breads, muffins, luncheon meats, cuts of meat and poultry, combination dishes). Results from the present study therefore suggest that the nutrient-specific system proposed by Health Canada might represent another effective way to influence the health outcomes of Canadians. However, this will remain hypothetical until modelling studies are conducted to specifically test the nutrient thresholds of Health Canada’s proposed system, which will most likely be based on pre-specified reference amounts and servings of stated size instead of per 100 g, as in TLL. Consumer testing will also remain imperative for such an approach to be successful, and the evaluation of how different sub-groups of the population (e.g. on the basis of age, gender, education, ethnicity, etc.) interpret the label would inform the development of appropriate and targeted education campaigns or tools that would support the implementation of this label.

A number of limitations and strengths need to be pointed out. First, cross-sectional NCD scenario models such as PRIME cannot incorporate the effect of time lag between exposure and disease outcome [13]. It is therefore not possible to clearly determine how long after the change in risk factor exposure the predicted changes in mortality from NCDs would occur. Also, the relative risk estimates used to parameterize the PRIME model were obtained from published meta-analyses which may not be the most current in the scientific literature at this point in time.

Second, the current TLL scenario modelled only one of the possible actual consumer responses to TLL, and it was not directly compared with other possible FOP labelling scenarios or with other types of nutrition-related policy-based interventions such as restricting the commercial marketing of unhealthy foods and beverages. However, this study represents the very first attempt to estimate the potential health impact of adopting a FOP labelling strategy in Canada, based on food consumption data from a nationally-representative nutrition survey and on the actual nutritional composition of Canadian-specific foods available in the country. In comparison, the only other study to date which modelled the potential health impact of FOP systems based on PRIME used food purchase data rather than food consumption data, in a sample that was not representative of the studied population [15].

Third, an “idealistic” TLL scenario was depicted here, in which it was assumed that in the context of a mandatory display of the traffic light symbols, all consumers would pursue a strategy of red avoidance whenever possible. As reported previously by our group [21], such a scenario is unlikely to occur in a real-life setting because other factors including individual preferences, nutrition knowledge, attitudes and behaviours, socio-cultural aspects and environmental determinants (e.g. price, availability, accessibility, convenience) are also known to influence food-selection decisions [40, 41]. This surely leads to an overestimation of the potential impact of the use of TLL by Canadians to improve their dietary intakes and health outcomes. It is however relatively difficult at the moment to design and test a mandatory FOP labelling scenario based on more realistic consumer behaviour given the paucity of evidence on this topic [42]. It is equally important to remember that not all food items carrying at least one red traffic light under the baseline scenario have been replaced by a comparable food without any red light under the TLL scenario. Despite our optimistic assumptions, over 75% of all food items carrying at least one red light under the baseline scenario actually remained unchanged under the TLL scenario, given the unavailability of comparable healthier alternatives on the market, which is not negligible. Moreover, it is expected that the introduction of a FOP label such as TLL could lead to product reformulation by the industry, primarily in order to remove red lights from products [17, 43], which we have not accounted for in the TLL scenario. Reformulation appears as an important channel by which FOP labels could beneficially impact dietary intakes of all strata of the population, irrespective of changes in consumer behaviour [6, 38].

Fourth, it is stressed that other positive changes might have occurred simultaneously in the intake of nutrients beyond those specifically considered in the TLL system, such as in mono- or polyunsaturated fat intakes, and that TLL would most likely exert potential benefits on morbidity in addition to reducing mortality, but these aspects were not accounted for in the present study.

## Conclusions

The present modelling study suggests that introducing in Canada a mandatory and interpretive nutrient-specific FOP labelling system such as TLL could be, if Canadians specifically tried to avoid foods labelled with red traffic lights, an effective population-wide intervention to improve NCD risk. Such data, supplemented with cost-effectiveness analyses, can support the adoption of nutrition-related public health strategies by government bodies.

## References

[1] Wartella EA, Lichtenstein AH, Boon CS. Examination of Front-of-Package Nutrition Rating Systems and Symbols: Phase I Report. Institute of Medicine (IOM). Washington, D.C.: The National Academies Press; 2010 [Cited 2016 November 21]. Available from: https://www.ncbi.nlm.nih.gov/books/NBK209847/pdf/Bookshelf_NBK209847.pdf 24983042

[2] Emrich TE, Qi Y, Mendoza JE, Lou W, Cohen JE, L'Abbe M R. Consumer perceptions of the Nutrition Facts table and front-of-pack nutrition rating systems. Appl Physiol Nutr Metab. 2014;39(4):417–24. 10.1139/apnm-2013-0304 24669982

[3] Gorton D, Ni Mhurchu C, Chen MH, Dixon R. Nutrition labels: a survey of use, understanding and preferences among ethnically diverse shoppers in New Zealand. Public Health Nutr. 2009;12(9):1359–65. 10.1017/S1368980008004059 19087382

[4] Feunekes GI, Gortemaker IA, Willems AA, Lion R, van den Kommer M. Front-of-pack nutrition labelling: testing effectiveness of different nutrition labelling formats front-of-pack in four European countries. Appetite. 2008;50(1):57–70. 10.1016/j.appet.2007.05.009 17629351

[5] Maubach N, Hoek J, Mather D. Interpretive front-of-pack nutrition labels. Comparing competing recommendations. Appetite. 2014;82:67–77. 10.1016/j.appet.2014.07.006 25038407

[6] Cecchini M, Warin L. Impact of food labelling systems on food choices and eating behaviours: a systematic review and meta-analysis of randomized studies. Obes Rev. 2016;17(3):201–10. 10.1111/obr.12364 26693944

[7] Hawley KL, Roberto CA, Bragg MA, Liu PJ, Schwartz MB, Brownell KD. The science on front-of-package food labels. Public Health Nutr. 2013;16(3):430–9. 10.1017/S1368980012000754 22440538 PMC10271311

[8] Egnell M, Talati Z, Hercberg S, Pettigrew S, Julia C. Objective Understanding of Front-of-Package Nutrition Labels: An International Comparative Experimental Study across 12 Countries. Nutrients. 2018;10(10). 10.3390/nu10101542 30340388 PMC6213801

[9] Ni Mhurchu C, Volkova E, Jiang Y, Eyles H, Michie J, Neal B, et al Effects of interpretive nutrition labels on consumer food purchases: the Starlight randomized controlled trial. Am J Clin Nutr. 2017;105(3):695–704. 10.3945/ajcn.116.144956 28148503

[10] Temple NJ. Front-of-package food labels: A narrative review. Appetite. 2019;144:104485 10.1016/j.appet.2019.104485 31605724

[11] Talati Z, Egnell M, Hercberg S, Julia C, Pettigrew S. Food Choice Under Five Front-of-Package Nutrition Label Conditions: An Experimental Study Across 12 Countries. Am J Public Health. 2019:e1–e6. 10.2105/AJPH.2019.305319 31622139 PMC6836805

[12] Kleef EV, Dagevos H. The growing role of front-of-pack nutrition profile labeling: a consumer perspective on key issues and controversies. Crit Rev Food Sci Nutr. 2015;55(3):291–303. 10.1080/10408398.2011.653018 24915389

[13] Scarborough P, Harrington RA, Mizdrak A, Zhou LM, Doherty A. The Preventable Risk Integrated ModEl and Its Use to Estimate the Health Impact of Public Health Policy Scenarios. Scientifica (Cairo). 2014;2014:748750 10.1155/2014/748750 25328757 PMC4195430

[14] Sacks G, Veerman JL, Moodie M, Swinburn B. 'Traffic-light' nutrition labelling and 'junk-food' tax: a modelled comparison of cost-effectiveness for obesity prevention. Int J Obes (Lond). 2011;35(7):1001–9. 10.1038/ijo.2010.228 21079620

[15] Egnell M, Crosetto P, d'Almeida T, Kesse-Guyot E, Touvier M, Ruffieux B, et al Modelling the impact of different front-of-package nutrition labels on mortality from non-communicable chronic disease. Int J Behav Nutr Phys Act. 2019;16(1):56 10.1186/s12966-019-0817-2 31307496 PMC6631735

[16] Scarborough P, Matthews A, Eyles H, Kaur A, Hodgkins C, Raats MM, et al Reds are more important than greens: how UK supermarket shoppers use the different information on a traffic light nutrition label in a choice experiment. Int J Behav Nutr Phys Act. 2015;12:151 10.1186/s12966-015-0319-9 26652916 PMC4676872

[17] Balcombe K, Fraser I, Falco SD. Traffic lights and food choice: A choice experiment examining the relationship between nutritional food labels and price. Food Policy. 2010;35(3):211–20. 10.1016/j.foodpol.2009.12.005

[18] Thorndike AN, Sonnenberg L, Riis J, Barraclough S, Levy DE. A 2-phase labeling and choice architecture intervention to improve healthy food and beverage choices. Am J Public Health. 2012;102(3):527–33. 10.2105/AJPH.2011.300391 22390518 PMC3329221

[19] Thorndike AN, Riis J, Sonnenberg LM, Levy DE. Traffic-light labels and choice architecture: promoting healthy food choices. Am J Prev Med. 2014;46(2):143–9. 10.1016/j.amepre.2013.10.002 24439347 PMC3911887

[20] Hieke S, Wilczynski P. Colour Me In—an empirical study on consumer responses to the traffic light signposting system in nutrition labelling. Public Health Nutr. 2012;15(5):773–82. 10.1017/S1368980011002874 22115180

[21] Emrich TE, Qi Y, Lou WY, L'Abbe MR. Traffic-light labels could reduce population intakes of calories, total fat, saturated fat, and sodium. PLoS One. 2017;12(2):e0171188 10.1371/journal.pone.0171188 28182630 PMC5300258

[22] Health Canada, Office of Nutrition Policy and Promotion, Health Products and Food Branch. Canadian Community Health Survey Cycle 2.2, Nutrition (2004): A Guide to Accessing and Interpreting the Data. Ottawa, Ontario: Health Canada; 2006 [Cited 2017 April 26]. Available from: http://www.hc-sc.gc.ca/fn-an/alt_formats/hpfb-dgpsa/pdf/surveill/cchs-guide-escc-eng.pdf

[23] Béland Y, Dale V, Dufour J, Hamel M. The Canadian Community Health Survey: Building on the Success from the Past. In: Proceedings of the American Statistical Association Joint Statistical Meeting, Section on Survey Research Methods, 2–6 August 2005. Minneapolis, MN: American Statistical Association; 2005. pp. 2738–2746.

[24] United States Department of Agriculture, Agricultural Research Service. AMPM—USDA Automated Multiple-Pass Method [Cited 2015 October 8]. Available from: http://www.ars.usda.gov/Services/docs.htm?docid=7710

[25] Health Canada. The Canadian Nutrient File. Nutrition Research Division. Ottawa (ON): Health Canada; 2015 [Cited 2018 February 26]. Available from: www.healthcanada.gc.ca/cnf

[26] United States Department of Agriculture, Agricultural Research Service. National Nutrient Database for Standard Reference Release 19. [Cited 2015 October 8]. Database: [Internet]. Available from: http://www.ars.usda.gov/ba/bhnrc/ndl

[27] Government of UK—Department of Health, Food Standards Agency, Welsh Government, The Scottish Government. Guide to Creating a Front of Pack (FoP) Nutrition Label for Pre-packed Products Sold through Retail Outlets. 2013 June [Cited 2015 February 19]. Available from: https://www.gov.uk/government/uploads/system/uploads/attachment_data/file/300886/2902158_FoP_Nutrition_2014.pdf

[28] Schermel A, Emrich TE, Arcand J, Wong CL, L'Abbe M R. Nutrition marketing on processed food packages in Canada: 2010 Food Label Information Program. Appl Physiol Nutr Metab. 2013;38(6):666–72. 10.1139/apnm-2012-0386 23724885 PMC4829389

[29] Statistics Canada. Table 051–0001—Estimates of population, by age group and sex for July 1, Canada, provinces and territories, annual (persons unless otherwise noted); 2016 [Cited 2016 January 27]. Database: CANSIM [Internet]. Available from: http://www5.statcan.gc.ca/cansim/a01?lang=eng

[30] Statistics Canada. Table 102–0522—Deaths, by cause, Chapter II: Neoplasms (C00 to D48), age group and sex, Canada, annual (number); 2016 [Cited 2016 January 29]. Database: CANSIM [Internet]. Available from: http://www5.statcan.gc.ca/cansim/a01?lang=eng

[31] Statistics Canada. Table 102–0524—Deaths, by cause, Chapter IV: Endocrine, nutritional and metabolic diseases (E00 to E90), age group and sex, Canada, annual (number); 2016 [Cited 2016 January 29]. Database: CANSIM [Internet]. Available from: http://www5.statcan.gc.ca/cansim/a01?lang=eng

[32] Statistics Canada. Table 102–0529—Deaths, by cause, Chapter IX: Diseases of the circulatory system (I00 to I99), age group and sex, Canada, annual (number); 2016 [Cited 2016 January 27]. Database: CANSIM [Internet]. Available from: http://www5.statcan.gc.ca/cansim/a01?lang=eng

[33] Statistics Canada. Table 102–0531—Deaths, by cause, Chapter XI: Diseases of the digestive system (K00 to K93), age group and sex, Canada, annual (number); 2016 [Cited 2016 January 29]. Database: CANSIM [Internet]. Available from: http://www5.statcan.gc.ca/cansim/a01?lang=eng

[34] Statistics Canada. Table 102–0534—Deaths, by cause, Chapter XIV: Diseases of the genitourinary system (N00 to N99), age group and sex, Canada, annual (number); 2016 [Cited 2016 January 29]. Database: CANSIM [Internet]. Available from: http://www5.statcan.gc.ca/cansim/a01?lang=eng

[35] *SAS* [computer program]. Version 9.3. Cary, NC: SAS Institute; 2011.

[36] *Software for Intake Distribution Estimation (SIDE)* [computer program]. Version 1.11. Ames, IA: Iowa State University; 2001.

[37] Statistics Canada. Canadian Community Health Survey (CCHS) Cycle 2.2 (2004) Nutrition–General Health (Including Vitamin & Mineral Supplements) & 24-Hour Dietary Recall Components: User Guide. Ottawa: 2008 April [Cited 2017 April 26]. Available from: http://www23.statcan.gc.ca/imdb-bmdi/document/5049_D24_T9_V1-eng.pdf

[38] Hawkes C, Smith TG, Jewell J, Wardle J, Hammond RA, Friel S, et al Smart food policies for obesity prevention. Lancet. 2015;385(9985):2410–21. 10.1016/S0140-6736(14)61745-1 25703109

[39] Health Canada. Toward Front-of-Package Nutrition Labels for Canadians: Consultation Document. 2016 [Cited 2017 February 22]. Available from: https://www.canada.ca/en/health-canada/programs/front-of-package-nutrition-labelling/consultation-document.html

[40] Raine KD. Determinants of healthy eating in Canada: an overview and synthesis. Can J Public Health. 2005;96 Suppl 3:S8–14, S8-5. 10.1007/BF03405195 PMC6976210 16042158

[41] Lee A, Mhurchu CN, Sacks G, Swinburn B, Snowdon W, Vandevijvere S, et al Monitoring the price and affordability of foods and diets globally. Obes Rev. 2013;14 Suppl 1:82–95. 10.1111/obr.12078 24074213

[42] Kanter R, Vanderlee L, Vandevijvere S. Front-of-package nutrition labelling policy: global progress and future directions. Public Health Nutr. 2018;21(8):1399–408. 10.1017/S1368980018000010 29559017 PMC10261279

[43] Grunert KG, Wills JM. A review of European research on consumer response to nutrition information on food labels. J Public Health. 2007;15:385–99. 10.1007/s10389-007-0101-9

